# Implementation of strength-based case management for opioid-dependent patients presenting in medical emergency departments: rationale and study design of a randomized trial

**DOI:** 10.1186/s13063-020-04684-6

**Published:** 2020-09-03

**Authors:** Amber Regis, Sarah E. Meyers-Ohki, Sarah E. Mennenga, Peter P. Greco, Richard Glisker, Rhonda Kolaric, Ryan P. McCormack, Richard C. Rapp, Michael P. Bogenschutz

**Affiliations:** 1 NYU Grossman School of Medicine, New York, USA; 2 Wright State University, Boonshoft School of Medicine, Dayton, OH USA

**Keywords:** Opioid epidemic, Opioid dependence, Emergency department, Brief interventions, Strength-based case management

## Abstract

**Background:**

As the USA grapples with an opioid epidemic, medical emergency departments (EDs) have become a critical setting for intervening with opioid-dependent patients. Brief interventions designed to bridge the gap from acute ED care to longer-term treatment have shown limited efficacy for this population. Strength-based case management (SBCM) has shown strong effects on treatment linkage among patients with substance use disorders in other healthcare settings. This study aimed to investigate whether SBCM is an effective model for linking opioid-dependent ED patients with addiction treatment and pharmacotherapy. Here, we describe the implementation and challenges of adapting SBCM for the ED (SBCM-ED). Study rationale, design, and baseline characteristics are also described.

**Methods:**

This study compared the effects of SBCM-ED to screening, assessment, and referral alone (SAR) on treatment linkage, substance use, and functioning. We recruited participants from a public hospital in NYC. Working alliance between case managers and participants and the feasibility of SBCM implementation were evaluated. Baseline data from the randomized sample were analyzed for group equivalency. Outcomes analyses are forthcoming.

**Results:**

Three hundred adult participants meeting DSM-IV criteria for opioid dependence were randomly assigned to either SBCM, in which they received a maximum of six case management sessions within 90 days of enrollment, or SAR, in which they received a comprehensive referral list and pamphlet outlining drug use consequences. No significant differences were found between groups at baseline on demographic or substance use characteristics. All SAR participants and 92.6% of SBCM-ED participants initiated their assigned intervention. Over half of SBCM-ED first sessions occurred in the ED on the day of enrollment. Case managers developed a strong working alliance with SBCM-ED participants after just one session.

**Conclusion:**

Interventions that exceed SBIRT were accepted by an opioid-dependent patient population seen in an urban medical ED. At the time of study funding, this trial was one of the first to focus specifically on this population in this challenging setting. The successful implementation of SBCM demonstrates its adaptability to the ED and may serve as a potential model for EDs seeking to adopt an intervention that overcomes the barrier between the ED encounter and more intensive treatment.

**Trial registration:**

ClinicalTrials.gov NCT02586896. Registered on 27 October 2015.

## Introduction

Opioid misuse is a critical public health emergency in the USA. According to the Substance Abuse and Mental Health Administration (SAMHSA), approximately 2.1 million Americans had opioid use disorder (OUD) in 2017 [1]. An estimated 1.7 million individuals within this group had prescription OUD, and approximately 700,000 had heroin use disorder [1]. According to the Centers for Disease Control and Prevention (CDC), the percentage of drug overdose deaths involving heroin tripled from 8% in 1999 to 25% in 2015 [2]. In 2016, opioids accounted for 42,249 (66.4% of total) drug overdose fatalities; in 2017, that number rose to 47,600 (67.8% of all drug overdose deaths) [3].

Escalating rates of opioid-related mortality can be curbed by effective treatments [4]. Studies show that OUD can be treated using medical models of care and pharmacotherapy [5–10]. However, according to the 2017 National Survey on Drug Use and Health, 18.2 million individuals identified as needing substance abuse treatment, including those with opioid dependency, had not received any inpatient or outpatient treatment services within the past 12 months [1].

Amid this national crisis, emergency departments (EDs) offer a vital intervention point for identifying patients with OUD and connecting them with treatment [11]. Individuals with OUD have increasingly sought the ED for problems related to opioid misuse, including withdrawal, injection-site abscesses, and opioid overdose [12]. In 2015, 140,077 ED visits were related to nonfatal opioid poisonings, including 81,326 visits related to heroin [13]. Between the third quarter of 2016 and the third quarter of 2017 alone, ED visits related to opioid overdose rose by 30% in all regions of the USA [3]. Despite these estimates, the ED remains underused as a setting for transitioning OUD patients to longitudinal treatment [11, 14].

### Screening, brief intervention, and referral to treatment (SBIRT) in the ED

One existing evidence-based intervention employed in the ED to address the health risks associated with substance abuse is screening, brief intervention, and referral to treatment (SBIRT) [15]. SBIRT was originally designed for intervening with primary care patients who use tobacco and alcohol, but has more recently been used in the ED, and with opioid-dependent patients. Typically, SBIRT staff use a standardized screening tool to identify at-risk patients or patients with problematic substance use. Patients are engaged in a brief conversation intended to help them understand the health consequences of their drug and alcohol use and motivate them to reduce or give up their use by encouraging more intensive treatment. Referral to treatment involves facilitating access to treatment services and establishing follow-up plans [16–18].

Although SBIRT has been endorsed by governmental and societal agencies such as the SAMHSA and the World Health Organization (WHO) [11], results from individual clinical trials and systematic reviews are equivocal regarding its efficacy in the ED setting [19]. While SBIRT has shown some efficacy with alcohol-dependent patients in the ED [20–22], research on its effects with patients who use other substances, such as opioids, is limited. One randomized controlled trial found that a brief ED intervention for patients with moderate to severe drug use did not improve outcomes relative to either minimal screening or screening, assessment, and referral [23]. The authors concluded that brief interventions were not sufficient to address the treatment needs of patients affected by severe drug problems and that more intensive ED interventions should be explored [23].

### Case management for patients with substance use disorders (SUDs)

Case management is widely used as an intervention for populations with SUDs and has been shown to improve linkage and engagement with substance abuse treatment and other ancillary services [24–26]. In studies with opioid-dependent populations, case management has been associated with improved linkage and retention in treatment among patients discharged from methadone maintenance programs [27, 28] and with injection drug users [29].

A 2007 Cochrane Systematic Review [26] examined five models of case management as used in randomized controlled trials with persons with SUDs: brief brokerage model that connects clients to services in one or two contacts; generalist/intensive assessment, planning, linking, monitoring, and advocacy; assertive community treatment involving a team of case managers who provide assertive outreach and direct counseling; clinical case management, which can include psychotherapy; and strength-based case management (SBCM), which incorporates a focus on strengths and other relationship-building activities to encourage continued participation with the intervention. Although the small number of published studies limited meta-analyses, the authors were able to compare the effect sizes of three models and found that SBCM had the highest effect, followed by brokerage and intensive case management models.

### SBCM

SBCM has been implemented in various health care settings, including hospitals, central intake units, and public health care locales [30–33]. The original clinical trials of long-term strength-based case management (up to 9 months) were implemented to improve retention among a primarily crack cocaine using population immediately following residential treatment at a Veterans Administration hospital [34]. Having a case manager and aftercare counselor compared to only an aftercare counselor predicted reduced levels of drug use at 6 months [35] and less criminal justice involvement at12 months [36]. Improvement in these critical outcomes was predicted by case management’s significant effect on retention in treatment: that is, patients with OUD who had case managers stayed in treatment longer and, as a result of staying in treatment, had better outcomes. Additionally, one third of the case management-aftercare group stopped their participation in aftercare but retained involvement with their case manager. The case management-only retainees demonstrated outcomes as favorable as clients who remained involved with aftercare [35].

SBCM was later adapted to a brief model to accommodate specific settings and goals. In a National Institute on Drug Abuse (NIDA)-funded clinical trial with SUD patients assessed at a centralized intake unit, five sessions of SBCM were compared to two sessions of motivational interviewing and a standard of care condition to determine SBCM’s efficacy in facilitating treatment linkage [31]. Patients who received SBCM linked with treatment 18% more often than the standard of care group, and the odds of linking with treatment were doubled (motivational interviewing had no discernible effect on linkage) [31]. The brief model of SBCM also demonstrated positive outcomes in a CDC-funded multi-site randomized controlled trial to improve linkage with care among individuals newly diagnosed with HIV [37]. Results from this study showed that participants assigned to SBCM were significantly more likely to have had at least one HIV primary care visit in each of 2 consecutive 6-month follow-up periods, as compared to the “passive referral” group (who received information about HIV and local care resources as well as a referral to a local HIV medical care provider). In a subsequent 10-site effectiveness study where program personnel were responsible for implementing SBCM without the rigorous controls of a clinical trial, 79% of participants visited an HIV clinician at least once within the first 6 months, similar to the results of the related clinical trial [38].

### Study rationale

As the rates of drug-related deaths and SUD-related emergency room visits continue to climb, attention must be paid to bridging the gap between the ED encounter and more intensive treatment. This area is of critical importance for patients with OUD, whose needs are not met by brief interventions or brief treatment, and for whom pharmacotherapy is often indicated [12]. Emergency room interventions for SUDs have largely been limited to SBIRT models, and these have focused primarily on alcohol. Although there is a substantial literature documenting the value of case management in linking patients with drug dependence to treatment, this approach has not been applied to patients in the ED setting.

This study aimed to investigate whether SBCM is an effective model for linking ED patients with OUD to longer-term addiction treatment. Taking into account the evidence-based research supporting pharmacotherapy for OUD, the SBCM model adapted for this study (herein referred to as SBCM-ED) emphasized linkage to effective pharmacotherapy (buprenorphine, methadone, or naltrexone) for patients who desired these treatments. SBCM-ED was implemented as a brief, but more intense, intervention that extends beyond the ED visit, as compared to other brief intervention or SBIRT models. At the time of study funding, it was one of the first trials to focus specifically on opioid-dependent patients seen in medical EDs.

Here, we discuss and describe SBCM-ED implementation and challenges as they relate to recruitment and intervention initiation. Given the increased number of individuals seeking EDs for opioid use-related problems, the successful implementation of this model demonstrates the capability of brief, but more intense ED intervention initiation in ED settings, and how the ED can be leveraged to connect patients with OUD to addiction treatment and pharmacotherapy. The study design, procedures, and baseline characteristics of the study sample are also described.

## Methods

### Design overview

This two-group randomized trial was funded by NIDA (Grant # R01DA034613). The specific aims of the study were to compare the effects of SBCM-ED vs. screening, assessment, and referral (SAR) alone on (1) initiation of addiction treatment and engagement in pharmacotherapy, (2) substance use, and (3) broader measures of health and life functioning, all at 3 months following an ED visit. Additional aims were to (4) examine the interactions between treatment assignment and selected participant attributes in predicting treatment initiation, pharmacotherapy engagement, and substance use outcomes and to (5) examine whether treatment initiation and pharmacotherapy engagement significantly mediate the effects of study interventions on substance use outcomes. The study was approved by an academic Institutional Review Board and the Office of Research Administration at the host site.

### Recruitment procedures

Participant recruitment occurred between March 2016 and October 2018 in the high-volume ED (approximately 125,000 ED visits annually; approximately 6600 SUD-related visits annually) of a public-sector hospital in New York City with a volunteer SBIRT program and limited ED social work department. Potential candidates were recruited following their registration in the ED. Candidates could be approached directly by study staff if their registered chief complaint indicated possible opioid dependence (e.g., “seeking detox from heroin,” “heroin overdose”), or be referred to the study by ED clinical staff or SBIRT volunteers when they encountered patients who screened positive for opioid use. Study staff obtained permission from providers before speaking with potential candidates and screening them for study eligibility.

### Inclusion and exclusion criteria

Participants were men and women presenting for medical treatment in the ED who met the following inclusion criteria: 18 years of age or older, proficient in English, registered as a patient in the ED during screening hours, endorsed at least three opioid-dependence criteria by self-report on the DSM-IV checklist, and self-reported misuse of opioids within the 30 days before screening. Candidates were excluded if they met any of the exclusion criteria: inability to participate due to emergency treatment, significant impairment of cognition or judgment rendering the person incapable of informed consent, status as a prisoner or in police custody, current engagement in substance use disorder treatment, inability to provide two reliable locators, unavailability for follow-up, prior participation in the current study, or current participation in a research study related to substance use.

### Screening and baseline procedures

Using a brief IRB-approved script, study staff approached potentially eligible patients and obtained their verbal consent to complete a pre-screen assessment. Preliminary, anonymous data were collected to determine potential eligibility and provide information on the representativeness of the study sample. First, a brief information tool recorded candidates’ chief complaint, triage acuity level, gender, age, prisoner status, English proficiency, and the number of days of opioid use within the 30 days before screening. If inclusion/exclusion criteria were preliminarily met, the DSM-IV checklist [39] was administered for each illicit substance used within the last 12 months. ED patients who met DSM-IV criteria for opioid dependency were screened for remaining eligibility criteria using a secondary screening form. Trained research staff conducted written informed consent with interested and eligible participants. Participants were then assigned a study ID and enrolled in the main study database.

### Study assessments

Study data were collected and managed using REDCap (Research Electronic Data Capture) electronic data capture tools hosted by the study’s academic institution [40, 41]. REDCap is a secure, web-based software platform designed to support data capture for research studies, providing an interface for validated data capture; audit trails for tracking data manipulation and export procedures; automated export procedures for data downloads to common statistical packages; and procedures for data integration and interoperability with external sources.

The Form 90-D [42], a reliable and abbreviated version of the Form 90 interview [43], was used as the primary measure of participant substance use, treatment participation, and engagement in pharmacotherapy. The Form 90-D incorporates timeline follow-back procedures and assesses route of administration and severity of use for all substances used. At baseline, the Form 90-D assessed the 30 days before consent and also collected data on age of first use and lifetime months of use for substances of abuse [42]. The Form 90-D was re-administered at each of the study follow-ups (3 and 6 months), and participants were asked to report on all treatment and substance use since the previous time point.

Four participant self-administered assessments were administered at baseline and follow-up: The self-report abbreviated WHO Quality of Life questionnaire (WHOQoL-Bref) [44], The Texas Christian University—Treatment Motivation Assessment (TCU-TMA) [45], Short Inventory of Problems (SIP-D) [46], and Barriers to Treatment Inventory (BTI) [47]. The WHOQoL-BRef is a validated measure of quality of life and functioning and assesses psychological, social, physical health, and environmental domains (higher scores denote a higher quality of life) [44]. The TCU-TMA, which has been shown to be effective at estimating treatment engagement [45], measures four domains: problem recognition, desire for change, treatment readiness, and treatment reluctance (Likert scale 1–5; higher mean scores correspond to higher motivation). The SIP-D is a validated, drug-specific form of the alcohol-specific SIP [43, 46] and measures the consequences of substance use in the 3 months before the assessment (Likert scale 1–4; item responses summed; higher scores indicate more problems). The BTI is a reliable and valid measure of treatment barriers (Likert scale 1–5; higher scores indicate more agreement that a given barrier is present), including lack of social support, fear of treatment, privacy concerns, and poor treatment availability [47]. Three items were added to the BTI to assess barriers to treatment associated with childcare, child custody, and child protective services.

A demographic form and a standard locator information form, to capture participants’ primary contact information and the contact information for at least two locators, were completed once at baseline (locator form was updated throughout the study).

### Randomization

Following the completion of baseline assessments and a final documentation of eligibility, participants were randomly assigned in a 1:1 ratio to either SBCM-ED or SAR. Randomization followed a randomly permuted block randomization schedule set up prior to the trial start and concealed from research staff. As studies are not consistent as to which factors may predict treatment engagement and opioid use outcomes, particularly in ED samples [48–51], randomization was not stratified. Trained research staff activated the allocation table by pushing a “randomize” button in the REDCap data capture system. Study participants and research staff delivering the study interventions were not blinded to the treatment assignment. A study staff member blinded to treatment assignment conducted 3 and 6-month follow-ups.

### Study interventions

#### SAR intervention

The SAR intervention represented a level of care significantly higher than treatment as usual in the ED. Immediately following randomization to the SAR group, participants were read a brief verbal script informing them that they met the criteria for OUD and should consider treatment. Participants received a 2-page informational pamphlet about the effects of substance use on the body and a 13-page referral list of over fifty SUD treatment options in the community, including detoxification services, outpatient services, residential/rehabilitation services, medication-assisted treatment programs, and peer support groups. This referral list was organized by borough and provided significantly more information and direction than the generic referral list provided in the ED. For each referral site, it included the phone number, location, website, hours of operation, insurance accepted, available pharmacotherapy, and information on how to begin the admissions process. Additionally, the referral list included smoking cessation resources, links to psychoeducation, and crisis hotline numbers. When presenting the referral list to participants, research staff explained the information contained therein and answered any general questions.

#### SBCM-ED intervention

Participants randomized to SBCM-ED were eligible to receive case management during the 90 days following randomization. Case managers aimed to conduct the initial SBCM-ED session as soon as possible following the participant’s randomization (and ideally, before the participant left the ED). Participants were offered a maximum of six sessions within 90 days, with the primary goal of facilitating linkage to treatment as soon as possible. If a participant linked with treatment, they were eligible, but not required, to attend up to two post-linkage sessions; however, no participant could exceed six sessions (pre-/post-linkage combined) during the 90-day period. Termination of the relationship occurred once 90 days had elapsed from the participant’s date of consent, or when the participant exhausted all possible sessions, or if the participant withdrew voluntarily.

#### SBCM-ED training

SBCM-ED case managers were Bachelor’s and Master’s-level research staff who were familiar with the OUD population, local OUD treatment options, and community resources. Case managers also participated in all aspects of the trial, including participant recruitment and follow-up visits with participants whose treatment assignment they were blind to. Case managers engaged in a series of training calls with the principal developer of SBCM for SUD clients, followed by a 2-day in-person training. The in-person training consisted of a basic introduction to the SBCM model, a thorough review of the intervention manual, role-play exercises, and mock case management sessions. Case managers were then required to complete training cases with at least two consenting pilot participants (for a total of not less than five sessions). These sessions were audio-recorded, reviewed, and rated for fidelity by the SBCM developer. Fidelity scores were determined by adherence to the three main intervention principles (strengths focus, individual-driven, and working alliance; 15-items, Likert scale 0–5) and adherence to the prescribed session structure (single item; Likert scale 0–5). Once completed, pilot cases were discussed in a supervision call with the SBCM developer. Upon meeting satisfactory fidelity to the model during training cases, SBCM-ED case managers received full certification and were permitted to conduct SBCM-ED with study participants. Data from the satisfactory pilot cases were included in the main trial analyses. Over the course of the study, six case managers received certification.

Each SBCM-ED session was audiotaped with participant consent and rated by the SBCM-ED developer for fidelity. Adherence to the intervention was maintained with bi-weekly telephone supervision calls with the SBCM-ED developer to review fidelity scores, discuss new cases, troubleshoot any issues, and further develop the case managers’ intervention skills. The SBCM-ED developer also conducted annual in-person refresher workshops at the study site.

#### SBCM-ED model

The SBCM model was adapted to the ED context and manualized by one of the original developers of SBCM. The structure of SBCM-ED followed the widely accepted case management functions of assessment, planning, linking, monitoring, and advocacy [52, 53], and the theory-driven principles of the strengths perspective [54]. Strength-based principles include an emphasis on client strengths, teaching clients a method for setting and completing goals, and developing a strong working alliance [54, 55]. The emphasis on client strengths was based on the work of Bandura who found that individuals were most likely to perform desired behaviors successfully if they could find precedent for those behaviors in their own experiences [56–58]. The importance of client-driven goal setting was established in earlier clinical trials of SBCM [54, 55, 59]. The working alliance between the case manager and client has been identified as an essential element of client success [60–62].

Assertive outreach and encouraging the use of informal resources were also essential components of SBCM-ED. Assertive outreach, including meeting participants outside the office setting, was used to eliminate physical barriers that could prevent engagement in the intervention. Meeting in public spaces also worked to decrease the impact of the hierarchical relationship between the case manager and client, thus increasing the likelihood of developing a positive working relationship. SBCM-ED case managers frequently conducted sessions at various locations, such as inpatient units, restaurants, outpatient clinics, medical office waiting rooms, parks, residential treatment programs, and correctional facilities. Case managers also made themselves available to attend appointments with clients as requested. Additionally, public transportation fare payment (i.e., NYC MetroCards) was offered to clients after each session to ease potential transportation barriers. Informal resources included referring clients to free/low-cost resources such as self-help groups, faith organizations, and leisure-related groups, which may not be offered by formal treatment programs but serve as a vehicle for integrating into the community. Case managers also provided clients with a Google Voice phone number where they could reach the case manager directly if needed.

Each session of SBCM-ED was guided by specific objectives that promoted linkage with substance abuse treatment services and continued involvement with the case manager. In the first session, participants were introduced to the principles of SBCM-ED and the focus on personal strengths and goals. Case managers were clear about the aim of the intervention (to help individuals link with substance use treatment if that is their goal), but stressed that the intervention is individual-directed, and no pressure would be exerted to convince participants to link. Case managers then worked with participants to complete a personal inventory of strengths and to identify specific examples of how they implemented each strength in the past.

Participants then completed a “Personal Goals Roadmap,” in which they identified their goals and worked with the case manager to outline the objectives, specific strategies, potential barriers to overcome, related strengths that would aid in accomplishing their goals, person(s) responsible for completing each action item, and a target date for accomplishing the desired goals. If a client identified linkage to treatment as a goal, linkage activities by the case manager included providing detailed information about specific programs, calling programs on behalf of the client, assisting with phone screenings for programs, troubleshooting insurance issues, arranging for pickups from the client’s location directly to a treatment program, accompanying clients to walk-in admissions, and being present at intake appointments.

Subsequent sessions were dedicated to promoting participant strengths, facilitating linkage to services, identifying new goals, and reducing barriers to existing goals. Post-linkage sessions were dedicated to monitoring early participation in treatment and promoting treatment retention.

#### Working Alliance Inventory—Short Revised (WAI-SR)

The WAI-SR was a 12-item scale used to assess the extent to which participants experienced the case manager and case management as helpful. The WAI-SR was completed following the first SBCM session, ideally within 24 h. Participants self-reported how frequently they agreed with statements related to their experience of the case manager using a 5-point Likert scale ranging from one (“seldom”) to five (“always”). The WAI-SR consists of three subscales: the Goal subscale, which addresses the extent to which case management goals were important, mutual, and capable of being accomplished; the Task subscale, which focuses on the participant’s agreement about the steps taken to help improve the client’s situation; and the Bond subscale, which measures mutual liking and attachment [63].

### Statistical methods

#### Power and sample size

The target sample size of 300 randomized participants (150 per group) was based on power analyses calculated for between-group contrasts pertaining to the most important outcome measures: linkage to addiction treatment (initiation of treatment and engagement in pharmacotherapy) and substance use. In planned pairwise contrasts, calculations assumed an attrition rate of 15% in each cell and were based on a desired power of .80. For investigating linkage to addiction treatment, effect size estimates were based on reported effects for case management relative to treatment as usual (TAU) or another less intensive treatment [26]. Assuming a two-tailed test, df = 1, power projections suggested that the proposed cell sizes after attrition and Bonferroni correction (*α* = .025) were sufficiently powered to detect a differential effect as small as *d* = .32.

Regarding substance use, studies contrasting the effectiveness of case management with that of treatment as usual or lower intensity treatments have yielded heterogeneous results. While the overall effect relative to TAU was small (*d* = .12), larger effects on drug use were found in three studies with above-average linkage effects (*d* = .33) [26]. In the single most relevant trial, a large (*n* = 711) study with an opioid-dependent sample, case management yielded an intermediate (*d* = .23) reduction in drug use relative to psychoeducation and drug counseling [26]. Assuming a two-tailed test, df = 1, *α* = .05, power projections suggested that the proposed cell sizes would allow detection of effects as small as *d* = .20 to reject the null hypothesis, an effect size that falls midway between small and moderate.

#### Preliminary analyses

To evaluate the representativeness of the study sample, the randomized and not-randomized samples were compared on demographic and substance use characteristics collected during preliminary screening (unprotected *t* tests and chi-square analyses). Next, baseline descriptive statistics (mean, standard deviation) were generated on an expanded set of demographic and drug use variables within the randomized sample. Unprotected *t* tests and chi-square analyses were performed to assess treatment group equivalency. Additional unprotected *t* tests assessed for differences between SBCM-ED and SAR participants on measures of quality of life and functioning (WHOQol-Bref), problems associated with drug use (SIP-D), perceived barriers to treatment (BTI), and treatment motivation (TCU-MA) at baseline. The three-factor WAI-SR was scored by factor and average scores were reported for the SBCM-ED group. Alpha level was set at *p* < 0.01 for all comparisons.

Analyses of follow-up attrition by group assignment and predictors of attrition are planned, as are analyses to examine the convergent validity of self-reported treatment engagement with clinic-verified treatment records and self-reported substance use with urine drug screen data.

#### Outcomes analyses

In future outcome analyses, logistic regressions and general linear models will be used to examine intervention effects at 3 months on treatment engagement, post-randomization opioid use, and general functioning. Additional planned analyses will examine how environmental instability and other participant attributes at baseline moderate intervention effects, as well as the extent to which initiation and engagement in treatment mediate the relationship between group assignment and substance use outcomes. All analyses will be conducted in SPSS 25.0 or higher.^34^

## Results

### Participant recruitment

**Fig. 1:**
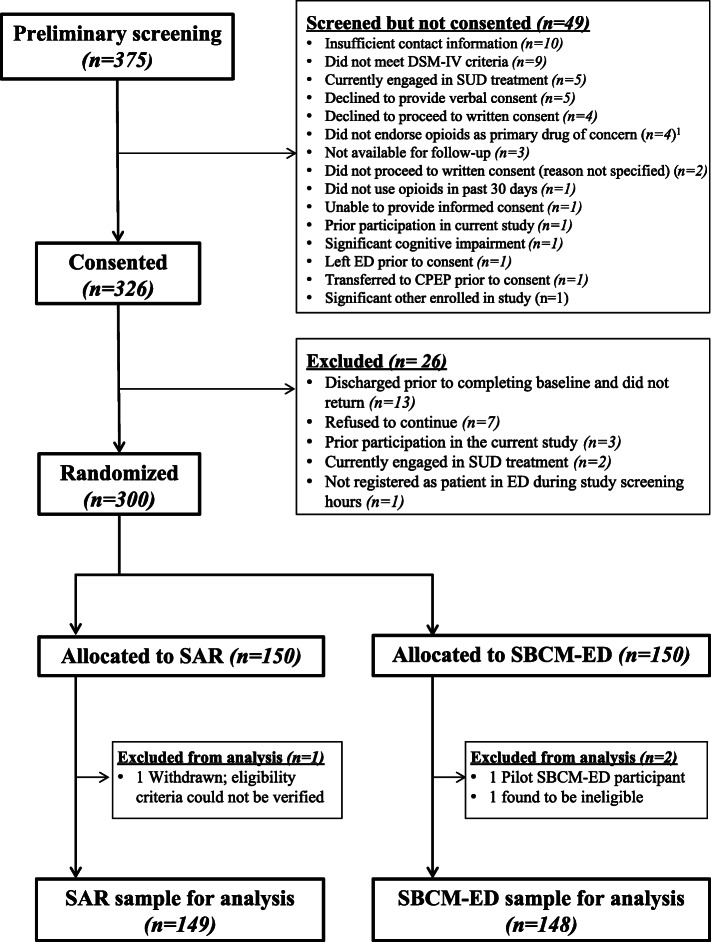
Participant flow from initial screening through randomization. ^1^Between April 2016 and August 2016, inclusion criteria required that opioids be the participant’s primary drug of concern. This criterion was modified to instead require that participants had misused opioids in the 30 days prior to consent

Recruitment began in March 2016 and concluded in October 2018. Figure 1 presents a diagram of the flow of patients through the trial. Verbal consent for preliminary, anonymous screening was obtained from 375 patients in the ED. Forty-nine patients did not proceed to consent, primarily because they did not have sufficient contact information or because they did not endorse three or more opioid-dependence criteria on the DSM-IV checklist. Of the 326 participants who completed written informed consented and proceeded to full screening, 26 were ultimately excluded, primarily because they were discharged from the ED prior to completing baseline and did not return to complete assessments. Three hundred participants were randomized (150 to each intervention). Three randomized participants were excluded from analysis: one was found to be ineligible following randomization, one was withdrawn because eligibility criteria could not be verified, and one participant was considered a pilot SBCM-ED participant (whose data was excluded because the case manager left the project before getting certified). The final sample for analysis includes 297 participants with 148 assigned to SBCM and 149 assigned to SAR.

### Demographics

Descriptive statistics (mean, standard deviation) on the characteristics of randomized participants are presented in Table 1 by group assignment. The randomized sample (*n* = 297) comprised 256 males (86.2%) and 41 females (13.8%) and ranged in age from 20.2 to 68.7 years (*M* = 43.7, *SD* = 10.8). The majority of participants identified as Hispanic/Latino (*n* = 98, 33.0%) or Black/African-American (*n =* 97, 32.7%). The sample was 24.9% White (*n* = 74); 2.7% Asian, Asian-American, or Pacific Islander (*n* = 8); and 6.7% identified as some other ethnic group (*n* = 20). The mean number of years of education was 11.7 (*SD* = 2.7). When asked whether or not they believed their living situation was stable and safe, 49.8% of participants said no and 50.2% said yes. No significant differences were found between groups at baseline. The randomized and non-randomized samples did not differ on demographic or substance use measures [64].

**Table 1:** Baseline demographics and drug use characteristics by treatment arm

	Total (***n*** = 297)		SAR (***n*** = 149)		SBCM-ED (***n*** = 148)	
Characteristic	***n***	***(%)***	***n***	***(%)***	***n***	***(%)***
Gender						
Male	256	(86.2)	126	(84.6)	130	(87.8)
Female	41	(13.8)	23	(15.4)	18	(12.2)
Age *mean years (SD)*	43.7	(10.8)	43.0	(10.8)	44.4	(10.8)
Race/ethnicity						
Hispanic/Latino	98	(33.0)	46	(30.9)	52	(35.1)
Black/African-American	97	(32.7)	50	(33.6)	47	(31.8)
White	74	(24.9)	38	(25.5)	36	(24.3)
Asian/Asian-Amer./Pacific Islander	8	(2.7)	5	(3.4)	3	(2.0)
Other	20	(6.7)	10	(6.7)	10	(6.8)
Income^1^ *mean (SD)*	15,788.70	(24,018.9)	15,581.97	(23,349.91)	15,942.49	(24,574.65)
Education *mean years (SD)*	11.7	(2.6)	11.8	(2.6)	11.5	(2.6)
Currently employed						
Yes	50	(16.8)	25	(16.8)	25	(16.9)
No	247	(82.3)	124	(83.2)	123	(83.1)
Stable living situation						
Yes	149	(50.2)	83	(55.7)	66	(44.6)
No	148	(49.8)	66	(44.3)	82	(55.4)
Lifetime opioid treatment (yes)	230	(77.4)	115	(77.2)	115	(77.7)
Chief complaint at ED visit						
Seeking detox	214	(72.1)	105	(70.5)	109	(73.6)
Withdrawal	17	(5.7)	5	(3.4)	6	(4.1)
Seeking medication/ methadone	11	(3.7)	8	(5.4)	9	(6.1)
Other	55	(18.3)	31	(20.8)	24	(16.2)
> 1 day(s) in healthcare institutions						
Hospital: medical	27	(9.1)	14	(9.4)	13	(8.8)
Hospital: detox	21	(7.1)	10	(6.7)	11	(7.4)
Residential detox	16	(5.4)	10	(6.7)	6	(4.1)
Ambulatory detox	2	(0.7)	2	(1.3)	0	(0.0)
Residential program	7	(2.4)	4	(2.7)	3	(2.0)
Residential: alcohol only	1	(0.3)	0	(0.0)	1	(0.7)
Residential: emotional/psych	5	(1.7)	2	(1.3)	3	(2.0)
Any institution	79	(26.6)	42	(28.2)	37	(25.0)
Incarcerated in past 30 days	15	(5.1)	5	(3.4)	10	(6.8)
Substance use^2^	***n***	***% or Mean (SD)***	***n***	***% or Mean (SD)***	***n***	***% or Mean (SD)***
Street opioid use						
Use in the last 30 (yes)	289	97.3%	146	98.0%	143	96.6%
Days in last 30	297	25.7 (7.9)	149	26.2 (7.2)	148	25.2 (8.6)
Lifetime months	293	185.3 (141.1)	147	179.4 (135.7)	146	191.3 (146.5)
Rx opioid use						
Use in the last 30 (yes)	93	31.3%	50	33.6%	43	29.1%
Days in last 30	297	2.6 (6.5)	149	2.74 (6.7)	148	2.5 (6.3)
Lifetime months	199	85.6 (109.4)	100	75.3 (93.8)	99	96.1 (122.7)
Alcohol use						
Use in the last 30 (yes)	165	55.6%	77	51.7%	88	59.5%
Days in last 30	297	10.2 (12.6)	149	9.5 (12.5)	148	10.8 (12.8)
Lifetime months	277	225.6 (149.4)	139	208.8 (142.6)	138	242.4 (154.6)
Cocaine use						
Use in the last 30 (yes)	193	65.0%	94	63.1%	99	66.9%
Days in last 30	297	9.4 (11.6)	149	9.3 (11.8)	148	9.5 (11.5)
Lifetime months	270	180.7 (144.0)	135	166.5 (138.3)	135	194.8 (148.7)

^1^Of those who reported income (*n =* 286)

^2^Results for most commonly used illicit substances in this sample

### Substance use

In the 30 days prior to enrollment, 289 participants (97.3%) reported using heroin and 93 participants (31.3%) reported using prescription opioids not as prescribed. Approximately 65.0% of the sample reported cocaine use and 55.6% reported alcohol use during the same period. Mean number of use days in the last 30 and number of lifetime months of use are presented for these substances in Table 1.

### Clinical and environmental characteristics

Table 1 also summarizes participants’ presenting or “chief complaints.” The primary chief complaint was, “seeking detox” (*n* = 214), followed by “other” (*n* = 55), “seeking medication/methadone” (*n* = 17), and complaints related to “withdrawal” (*n* = 11). During the 30 days prior to the index ED visit, 26.6% of the sample had received one or more days of SUD treatment (outpatient, residential, or prescribed medication) or spent one or more days in healthcare institutions (hospital, detox, residential detox, mental health institutions, etc.).

Average scores across factor groups on the participant self-report measures are presented in Table 2 (mean, standard deviation). Raw scores on the four WHOQol-Bref domains and separate total quality of life and total health scores were transformed to range between 4 and 20, comparable with the WHOQOL-100.

**Table 2:** Results of self-report measures, by factor

	Total (***n*** = 297)	SAR (***n*** = 149)	SBCM-ED (***n*** = 148)
	***Mean (SD)***	***Mean (SD)***	***Mean (SD)***
WHOQoL-Bref			
Physical	11.6 (2.9)	11.6 (2.9)	11.6 (2.9)
Psychological	10.9 (3.4)	10.9 (3.4)	10.9 (3.5)
Social	10.7 (3.9)	10.9 (3.9)	10.5 (3.8)
Environment	10.7 (3.1)	10.9 (3.2)	10.5 (3.1)
Total Quality of Life	9.3 (4.1)	9.2 (4.0)	9.4 (4.2)
Total Health	9.6 (4.1)	9.6 (4.0)	9.5 (4.2)
SIP-D			
Intrapersonal	10.9 (1.8)	10.8 (1.8)	10.9 (1.7)
Interpersonal	10.5 (2.1)	10.5 (2.1)	10.5 (2.1)
Physical	9.9 (2.1)	9.9 (2.0)	10.1 (2.1)
Social	11.1 (1.5)	10.9 (1.7)	11.2 (1.4)
Impulse Control	9.2 (2.4)	9.3 (2.5)	9.1 (2.3)
BTI			
Absence of Problem	1.7 (0.7)	1.6 (0.7)	1.7 (0.7)
Negative Social Support	1.8 (0.7)	1.8 (0.7)	1.8 (0.7)
Fear of Treatment	1.9 (0.7)	1.9 (0.7)	1.9 (0.8)
Privacy Concerns	2.6 (1.1)	2.7 (1.1)	2.6 (1.0)
Time Conflicts	2.0 (0.8)	2.0 (0.8)	2.0 (0.7)
Treatment Availability	2.3 (1.0)	2.3 (1.0)	2.3 (1.0)
Admission Difficulty	2.4 (1.0)	2.4 (0.9)	2.5 (1.0)
Childcare Concerns	1.8 (0.8)	1.8 (0.8)	1.7 (0.8)
TCU-MA			
Problem Recognition	4.1 (0.6)	4.1 (0.6)	4.2 (0.5)
Desire For Help	4.3 (0.5)	4.2 (0.5)	4.3 (0.5)
Treatment Readiness	3.9 (0.6)	3.9 (0.6)	4.0 (0.6)
External Pressure	2.9 (0.7)	2.9 (0.6)	2.9 (0.7)

### Intervention initiation

All of the participants randomized to SAR (*n* = 149) received the informational brochure and referral list. Of the 148 participants in the SBCM-ED sample analyzed, 137 (92.6%) engaged in at least one session. Table 3 shows that the majority of first sessions took place in the ED setting (*n* = 75, 54.7%). Several first sessions occurred on inpatient units within the hospital and in the case manager’s office (also on site). Of the 137 participants who completed session one, 112 (81.8%) completed the WAI-SR within 24 h of their session. Average scores on the WAI-SR for this group are as follows (*n*, mean, SD): Bond (*n* = 112, *M* = 4.5, *SD* = 0.1), Task (*n* = 112, *M* = 4.3, *SD* = 0.1), and Goal (*n* = 111, *M* = 4.4, *SD* = 0.1). These scores indicate that case managers and clients developed a high level of agreement on the tasks and goals of SBCM, as well as a strong affective bond [65, 66].

**Table 3:** SBCM-ED first session attendance, location, and working alliance

	SBCM-ED (***n*** = 148)	
	***n***	***(%)***
Session attendance		
0 sessions	11	(7.4)
> 1 session	137	(92.6)
Session 1 location		
Emergency department	75	(54.7)
Hospital (inpatient)	27	(19.7)
Case manager’s office	25	(18.2)
SUD treatment provider	2	(1.5)
Participant’s residence	1	(0.7)
Medical care clinic in hospital	2	(1.5)
Public location	3	(2.2)
Telephone	0	(0.0)
Car/Vehicle	1	(0.7)
Other	1	(0.7)
WAI-SR	***n***	***Mean (SD)***
Bond	112	4.5 (0.1)
Task	112	4.3 (0.1)
Goal	111	4.4 (0.1)

## Discussion

In the context of an unrelenting opioid epidemic and escalating rates of opioid-related ED visits, feasible interventions that go beyond a single brief encounter to link patients with effective long-term treatment and pharmacotherapy are needed [23]. The aim of this study was to evaluate the effects of a SBCM model on linkage, substance use, and functioning outcomes in an opioid-dependent population presenting to a medical ED. Testing models that go beyond other brief interventions, such as SBIRT, is in keeping with mixed results in the literature on brief interventions for patients with OUD and severe drug dependency.

Given the busy nature of the ED setting, flexibility of case managers and collaboration with ED staff/SBIRT volunteers was key to the successful implementation of this protocol. The ED setting required that case managers remain alert, adaptive, and mindful not to interrupt the flow of patient care. Depending on the participant’s triage level and location, study interventions were implemented in a variety of settings ranging from quiet private rooms to noisy bedside locations. When there was no designated area for treatment, case managers had to be extra mindful about protecting client confidentiality. ED staff were kept informed regarding the location of enrolled participants so that treatment was not delayed or derailed due to study involvement. We also found that open communication with SBIRT volunteers was crucial for achieving recruitment targets. The study was designed to leverage, in a pragmatic way, the existing ED flow of SBIRT screening and provider assessment: as noted, patients who screened positive on SBIRT measures of opioid misuse were referred to research staff. This referral model not only contributed to strong study recruitment, but also has real-world implications for how EDs can efficiently identify potential candidates for more intensive interventions like SBCM.

The demographics of the randomized sample reflect the general population of SUD patients seen by the study site, a large public hospital in New York City serving an ethnically diverse population of lower socioeconomic status. Within this sample of opioid-dependent patients, the screening process did not appear to favor any demographic characteristics other than the inclusion criteria, which limited participation to those not currently in treatment. However, the sample could reasonably be characterized as “treatment seeking” given that the majority presented with a chief complaint related to “seeking detox” and baseline inventory measures showed high levels of treatment readiness across groups. This quality may have contributed to the strong uptake of study interventions—100.0% in the SAR group and 92.6% in the SBCM-ED group.

Notably, the majority of initial SBCM-ED sessions took place, as intended, in the ED. Substance use patterns at baseline indicate that most participants were using opioids daily, and at the time of enrollment, many participants had presented to the ED in withdrawal from opioids and/or other substances. Withdrawal symptoms associated with opioid-dependency include dysphoric mood, nausea or vomiting, muscle aches, lacrimation or rhinorrhea, yawning, fever, and insomnia [39]. Other patients presented to the ED for overdose or abscess problems that required substantial medical stabilization and pain management. A barrier to implementation was that these types of symptoms and discomfort can compromise patients’ concentration, mood, and potentially, willingness to engage in drawn-out discussions of strengths and goals. Additionally, we observed that lengthy wait times (typical in EDs) could present a challenge to implementation, as patients may not be interested in meeting with the case manager after several hours already spent in the ED. In our study, 11 study participants did not complete session one, primarily because they left the ED before engaging with their assigned case manager.

Despite these challenges, study case managers were able to establish a strong working alliance with SBCM-ED participants after a single session, as evidenced by WAI-SR scores [65]. It may be relevant to note that study case managers had previous experience working with an OUD population, were familiar with the discomforts of withdrawal, and were trained to keep patients engaged by offering snacks and pausing for breaks.

### Study limitations

This study may be limited by the lack of a “treatment-as-usual” comparison group. However, for ethical reasons, a comparative effectiveness design was selected to ensure that participants not randomized to case management received referral information in a systematic process that sometimes exceeded the standard care. At the time of study initiation, there was no standard ED “treatment-as-usual” for opioid-dependent patients at the study site. For patients “seeking detox” (the majority of our sample), admission to inpatient stabilization depended on bed availability and patient eligibility for admission: patients were considered ineligible for detox if their last discharge from an inpatient or correctional facility was less than 30 days before their ED visit, if opioids were not present in their urine, or if they required a higher level of care for medical stabilization. Those not admitted were typically given a brief list of treatment providers in the community and a hand-off to social work for potential referrals to treatment programs and other social services. In some cases, patients presenting for non-opioid-related problems (e.g., injuries, x-rays) received medical care only and were not referred to SUD-specific treatment by clinical staff. Finally, the ED SBIRT volunteer program aimed to screen all patients presenting to the ED and provide naloxone kits to all patients with OUD; however, due to high patient volume, universal screening and brief intervention were not always possible. Participation in the study (for either group) did not preclude participants’ receiving whatever clinical care would otherwise be provided by the ED. Rather than alternatives to the standard of care, study interventions were considered additional treatments that may complement, or supplement, the suite of services already available.

A further possible limitation is that larger trends in ED treatment of OUD were not systematically tracked over the project’s 3-year recruitment period, so the impact of recent policy changes (for example, a 2018 campaign to increase buprenorphine prescribing in the ED) cannot be assessed. The project did abstract information from participants’ medical records regarding services received during the index ED visit (including pharmacotherapy administered in the ED), and these data will be considered during outcomes analyses.

## Conclusion

EDs are a vital conduit through which many individuals with OUD pass. Historically, interventions implemented in the ED, such as SBIRT, have been brief and may lack the clinical impact necessary to intervene in the multifaceted barriers facing individuals with OUD. Though the ED can be a challenging setting for intervening with OUD patients, early results from the implementation of SBCM-ED described here suggest that it was compatible with the ED setting and was accepted by patients with OUD. This demonstrates the potential for replication in other urban medical EDs, and that it is possible to conduct more intense ED interventions by leveraging existing resources, like SBIRT.

## Data Availability

The datasets used and/or analyzed during the current study are available from the corresponding author on reasonable request.

## Abbreviations

BTI: Barriers to Treatment Inventory
CDC: Centers for Disease Control and Prevention
ED: Emergency department
NIDA: National Institute on Drug Abuse
OUD: Opioid use disorder
REDCap: Research Electronic Data Capture
SAMHSA: Substance Abuse and Mental Health Administration
SAR: Screening, assessment, and referral
SBCM: Strength-based case management
SBCM-ED: Strength-based case management for the emergency department
SBIRT: Screening, brief Intervention, and referral to treatment
SIP-D: Short Inventory of Problems
SUD: Substance use disorder
TAU: Treatment as usual
TCU-TMA: The Texas Christian University—Treatment Motivation Assessment
WHOQoL-Bref: Abbreviated World Health Organization Quality of Life questionnaire
WAI-SR: Working Alliance Inventory—Short Revised
